# AOPs-SVM: A Sequence-Based Classifier of Antioxidant Proteins Using a Support Vector Machine

**DOI:** 10.3389/fbioe.2019.00224

**Published:** 2019-09-18

**Authors:** Chaolu Meng, Shunshan Jin, Lei Wang, Fei Guo, Quan Zou

**Affiliations:** ^1^College of Intelligence and Computing, Tianjin University, Tianjin, China; ^2^College of Computer and Information Engineering, Inner Mongolia Agricultural University, Hohhot, China; ^3^Department of Neurology, Heilongjiang Province Land Reclamation Headquarters General Hospital, Harbin, China; ^4^College of Computer Engineering and Applied Mathematics, Changsha University, Changsha, China; ^5^Institute of Fundamental and Frontier Sciences, University of Electronic Science and Technology of China, Chengdu, China; ^6^Center for Informational Biology, University of Electronic Science and Technology of China, Chengdu, China

**Keywords:** antioxidant proteins, machine-learning, sequence features, support vector machine, classifier

## Abstract

Antioxidant proteins play important roles in countering oxidative damage in organisms. Because it is time-consuming and has a high cost, the accurate identification of antioxidant proteins using biological experiments is a challenging task. For these reasons, we proposed a model using machine-learning algorithms that we named AOPs-SVM, which was developed based on sequence features and a support vector machine. Using a testing dataset, we conducted a jackknife cross-validation test with the proposed AOPs-SVM classifier and obtained 0.68 in sensitivity, 0.985 in specificity, 0.942 in average accuracy, 0.741 in MCC, and 0.832 in AUC. This outperformed existing classifiers. The experiment results demonstrate that the AOPs-SVM is an effective classifier and contributes to the research related to antioxidant proteins. A web server was built at http://server.malab.cn/AOPs-SVM/index.jsp to provide open access.

## Introduction

The antioxidant system in organisms has the ability to prevent damage caused by reactive oxygen species (ROS) (Siswoyo et al., 2011). The ROS, which include hydrogen peroxide, singlet oxygen, superoxide anion radical, hydroxyl radical, and nitric oxide, are the product of the metabolism and influence fatty acids, proteins, and DNA (Sögüt et al., 2003). An excess of ROS or the depression of the antioxidant system can lead to oxidative stress (Zima et al., 2001; Krishnaiah et al., 2007). This oxidative stress may then go on to lead to a series of pathological conditions such as heart disease, malaria, neurodegenerative diseases, AIDS, cancer, and the aging process (Ames, 1983; GEY, 1990; Ames et al., 1993; Smith et al., 1996; Diaz et al., 1997; Yang et al., 2019a).

Natural antioxidants are regarded as the second antioxidant defense line in organisms (Yigit et al., 2014), and have recently attracted increasing attention from researchers. Such antioxidants are mainly extracted from dietary sources such as fruits, vegetables, and foods with carotenoids and vitamin A (Geetha et al., 2002; Podsedek, 2007; Tang et al., 2019a,b). When these antioxidants are consumed, they scavenge from the ROS and minimize the oxidative stress, thus reducing the risk to organisms (Yang et al., 2017). Many extracted or purified proteins are used as natural antioxidants, including soy proteins, lactoferrin, casein, β-lactoglobulin, canola proteins, yam dioscorin, egg albumen proteins, maize zein, egg yolk phosvitin, and potato patatin. In addition, proteins extracted from fertilized eggs, jellyfish, white beans, chickpeas, melinjo (gnetum gnemon) seeds, and ginkgo biloba seeds were also reported to have antioxidant properties (Rajalakshmi and Narasimhan, 1996; Chiue et al., 1997; Maheswari et al., 1997; Kouoh et al., 1999; Satué-Gracia et al., 2000; Hou et al., 2001; Liu et al., 2003; Cumby et al., 2008; Huang et al., 2010; Li et al., 2017). *In vitro* assay systems are commonly employed to identify the antioxidant activity of a new protein, including any scavenging effect on DPPH and ABTS, the inhibition of linoleic acid autoxidation, any chelating or strength-reducing capabilities, and protections against DNA damage caused by hydroxyl radical-mediation (Liu et al., 2003; Dastmalchi et al., 2008; Sachindra and Bhaskar, 2008; Huang et al., 2010; Fu et al., 2018). However, the *in vitro* experiment is time-consuming and inefficient. Therefore, to increase the success rate, it is desirable to develop a classifier to confirm antioxidant proteins prior to the *in vitro* experiment.

Recently, several researchers have used a computational approach to the identification of antioxidant proteins. Enrique Fernandez-Blanco et al. used star graph topological indices and random forests to develop a model for identifying antioxidant proteins (Fernández-Blanco et al., 2013). However, when analyzing the dataset, we found that the sequences used for the training model do not include the removal of redundant data. As a result, data similarity increases, which makes the results of the model untrustworthy. In 2013, Feng et al. developed a Naive Bayes model based on a sequence feature (Feng et al., 2013b), and in 2016, they constructed a model named AodPred based on the support vector machine using a 3-gap dipeptides feature (Feng et al., 2016). Xu et al. also used the support vector machine to construct a model to identify antioxidant proteins (Xu et al., 2018). The latter two models were built on the same training dataset and included a sequence to remove redundant data. The analysis of the results indicates that there is room to improve the identification accuracy. The training set for our model is the same as the two models mentioned above. In the bioinformatics field, applying computational methods to identify a particular protein mainly requires machine-learning techniques. The process can be divided into two main steps: (1) extracting features from protein sequences, and (2) constructing classifiers.

The first step is to extract discriminative features from a protein sequence. Sequence-order information or its combination with biochemical characteristics of proteins is a common approach. The most popular is the pseudo amino acid (PseAAC) method proposed by Shen and Chou (2006). Subsequently, many methods based on PseAAC have emerged (Liu et al., 2015, 2017; Zhu et al., 2015, 2018; Chen et al., 2016; Tang et al., 2016; Yang et al., 2016). In addition, there are also features to indicate the evolutionary and secondary structure information, primarily the PSI-BLAST (Altschul et al., 1997) and PSI-PRED (Jones, 1999) profiles. Then, a dimension-reduction algorithm is often applied to reduce the redundant information of extracting features (Liu, 2017; Tang et al., 2018; Xue et al., 2018; Tan et al., 2019; Zhu et al., 2019); these include ANOVA (Anderson, 2001; Ding and Li, 2015; Li et al., 2019b), mRMR (Peng et al., 2005), and MRMD (Zou et al., 2016b). These algorithms rank the features using certain criteria and then select the optimal feature. In the second step, classification algorithms have been applied to train on the optimal feature set and construct model. The support vector machine has been widely used and has obtained good results (Ding and Dubchak, 2001; Shamim et al., 2007; Yang and Chen, 2011; Feng et al., 2013a; Zou et al., 2016a; Ding et al., 2017; Chen et al., 2019). Furthermore, other classification methods, such as the hidden Markov mode (Bouchaffra and Tan, 2006), random forests (Dehzangi et al., 2010), and neural networks (Chen et al., 2007) have been used in this step. There are also ensemble classifiers. For example, Zou et al. proposed libD3C (Lin et al., 2014), which integrates multiple weak classifiers and voting for the final result.

## Materials and Methods

### Benchmark Dataset

We used the same dataset as Feng and Xu et al. The positive dataset was generated as follows. (1) The sequences marked as “antioxidant” in the Universal Protein Resource (Uniport) (2014_02 release) were selected. (2) Sequences that contained residues such as “B,” “X,” and “Z,” were eliminated because of their uncertain meaning. (3) The protein sequences labeled with “reviewed” were the only ones considered to ensure that the selected sequences had been verified through experiments. The negative dataset was constructed with a list of PISCES-culled PDB (Wang and Dunbrack, 2003) proteins with identification values <20%, in the same manner as Fernández-Blanco et al. (2013). These steps resulted in 710 positive samples and 1,567 negative samples. To avoid a low quality dataset that may incorrectly predict the result, the CD-HIT program (Fu et al., 2012) was applied with a 60% threshold to obtain a benchmark dataset. This final dataset included 253 antioxidant proteins and 1,552 non-antioxidant proteins, which can be expressed as follows:

(1) $$\begin{array}{l} Set={Set}_{+}\cup {Set}_{-} \end{array}$$

Where *Set*_+_ represents the positive dataset (the 253 antioxidant proteins); *Set*_−_ represents the negative dataset constructed from 1,552 non-antioxidant proteins; and the “∪” symbol indicates that the benchmark dataset consisted of positive and negative datasets. The proportion of positive and negative samples is ~1:6, which represents an unbalanced dataset.

### Feature Extraction

In this study, we used the feature extraction algorithm (abbreviated as 473D) proposed by Wei et al. (2015). This algorithm generates 473 discrete features based on the PSI-BLAST (Altschul et al., 1997) and PSI-PRED (Jones, 1999) profiles. The former contains the evolutionary information and the latter contains the secondary structure information of the protein sequence. First, a protein with a number of amino acid residues is defined as:

(2) $$\begin{array}{l} S=A_{1}A_{2}A_{3}\ldots A_{\text{n}-1}A_{\text{L}} \end{array}$$

where *A*_*i*_ means the *i*th amino acid residue of a protein sequence. Then, the 473D feature is extracted from the protein sequence in the following steps.

(1) Extract 20 features from a position-specific score matrix (PSSM) (Xiong et al., 2018). The PSSM is a matrix generated by running the PSI-BLAST program on a protein sequence of length, which is represented as (Wei et al., 2015)

(3) $$\begin{array}{l} M_{PSSM}={\left|\begin{array}{cccc} p_{1,1} & p_{1,2} & \cdot \cdot \cdot & p_{1,20}\\ p_{2,1} & p_{2,2} & \cdot \cdot \cdot & p_{2,20}\\ \vdots & \vdots & \vdots & \vdots \\ p_{i,1} & p_{i,2} & \cdot \cdot \cdot & p_{i,20}\\ \vdots & \vdots & \vdots & \vdots \\ p_{L,1} & p_{L,2} & \cdot \cdot \cdot & p_{L,20} \end{array}\right|}_{L\times 20}s.t.1\leq i\leq L \end{array}$$

where each PSSM matrix entry is equal to the muting score of the *i*th amino acid residue in protein sequence *S* and the *n*th amino acid residue in the amino acid alphabet. The value of entries in *M*_*PSSM*_ are grouped by the same column and averaged to form 20 values. Then, they are combined to generate a vector *F*_*pssm*_ with a length of 20, which can be formulated as follows (Wei et al., 2015):

(4) $$\begin{array}{lll} {\text{F}}_{\text{p}\text{s}\text{s}\text{m}} & = & \{\left({\text{f}}_{1},{\text{f}}_{2},{\ldots ,{\text{f}}_{\text{n}},\ldots ,\text{f}}_{20}\right)|{\text{f}}_{\text{n}}\\ \; & = & \frac{1}{\text{L}}\sum_{\text{i}=1}^{\text{L}}{\text{p}}_{\text{i},\text{n}}\;\text{a}\text{n}\text{d}\;1\leq \text{n}\leq 20\} \end{array}$$

where *f*_*n*_ equal to the average score of each residue in the sequence *S*, mutating to *n*th amino acid residue in the evolutionary process.

(2) Extract 20 one-gram and 400 two-gram features from the frequency matrix. Each entry in the PSSM matrix multiplied by the corresponding background frequency is taken as the exponent and two (2) is the base. Then, the frequency matrix is obtained by a power operation as follows (Wei et al., 2015):

(5) $$M_{\text{frequency}}={\left|\begin{array}{cccc} 2^{p_{1,1}\times bf_{1}} & 2^{p_{1,2}\times bf_{2}} & \cdot \cdot \cdot & 2^{p_{1,20}\times bf_{20}}\\ 2^{p_{2,1}\times bf_{1}} & 2^{p_{2,2}\times bf_{2}} & \cdot \cdot \cdot & 2^{p_{2,20}\times bf_{20}}\\ \vdots & \vdots & \vdots & \vdots \\ 2^{p_{\text{i},1}\times bf_{1}} & 2^{p_{\text{i},2}\times bf_{2}} & \cdot \cdot \cdot & 2^{p_{\text{i},20}\times bf_{20}}\\ \vdots & \vdots & \vdots & \vdots \\ 2^{p_{\text{L},1}\times bf_{1}} & 2^{p_{\text{L},2}\times bf_{2}} & \cdot \cdot \cdot & 2^{p_{\text{L},20}\times bf_{20}} \end{array}\right|}_{L\times 20}s.t.1\leq \text{i}\leq L$$

where *M*_*frequency*_ is the frequency matrix, *p*_*i,n*_ is the PSSM *i*th row and *n*th column entry, and *bf*_*j*_ is the background frequency of amino acid in the amino acid alphabet (The value of *bf*_*j*_ is provided on the website http://server.malab.cn/AOPs-SVM/data.jsp). The consensus sequence is generated from the first row to *L*th row of *M*_*frequency*_ per the following criteria. To *i*th row of the *M*_*frequency*_, determine the largest entry $2^{p_{i,j}\times bf_{j}}\;$ according to its column order, and choose the *j*th amino acid in the amino acid alphabet. Repeat this step *L* times to generate a new consensus sequence *S*_*c*_. From the analysis of the above process, it is concluded that *S*_*c*_ is the evolutionary result of *S*, because each amino acid residue in *S* is replaced by the most frequent amino acid to generate *S*_*c*_. Then, a one-gram and two-gram algorithm are used to extract the frequency of occurrence features from the sequence *S*_*c*_. The one-gram algorithm calculates the frequency of 20 amino acids residue in the sequence, and the two-gram algorithm calculates the frequency of 20 × 20 possible amino acid residue adjacent pairs in the sequence, which are represented by (Wei et al., 2015):

(6) $$\begin{array}{lll} F_{1-gram} & = & \{\left(f_{1},f_{2},{\ldots ,f_{n},\ldots ,f}_{20}\right)|f_{n}\\ \; & = & \frac{1}{L}O\left(A_{i}\right)\;and\;1\leq i\leq 20\;\} \end{array}$$

(7) $$\begin{array}{lll} F_{2-gram} & = & \{\left(f_{1},f_{2},{\ldots ,f_{n},\ldots ,f}_{20\times 20}\right)|f_{n}\\ \; & = & \frac{1}{L-1}O\left(A_{i}A_{j}\right)\;and\;1\leq i,j\leq 20\} \end{array}$$

where *O*(*x*) means the occurrence time of *x*, *A*_*j*_ is the amino acid alphabet, which can be a single amino acid residue *A*_*i*_ or amino acid residue adjacent pair *A*_*i*_*A*_*j*_, and *L* is the sequence length. Then, by proportionally weighting *F*_1−*gram*_ and *F*_2−*gram*_, the 420 features are obtained, which are represented as (Wei et al., 2015)

(8) $$\begin{array}{l} \left\{F^{\prime }{\;}_{1-gram},F^{\prime }{\;}_{2-gram}\right\}=\left\{F_{1-gram}\times \frac{20}{420}\;,F_{2-gram}\times \frac{400}{420}\right\} \end{array}$$

(3) Extract six features from the PSI-PRED secondary structure sequence. Program PSI-PRED can generate a secondary structure sequence *S*_*structure*_ from protein sequence *S*, which is represented as (Wei et al., 2015):

(9) $$\begin{array}{lll} S_{structure} & = & T_{1}T_{2}T_{3}\ldots T_{i}\ldots T_{L-1}T_{L}\;s.t.T_{i}\in \left\{H,E,C\right\}\;and\\ \; & \; & 1\leq i\leq L \end{array}$$

where *H*, *E*, and *C* represent the secondary structure states of helix, strand, and coil, respectively. This means that the secondary structure sequence *S*_*structure*_ is generated by each amino acid residue in protein sequence replaced by one of letter in *H*, *E*, and *C*. Then, from the sequence *S*_*structure*_, extract five features as follows (Wei et al., 2015):

(10) $$\begin{array}{l} F_{H}=\frac{\sum_{i=1}^{{Count}_{h}}{Posi}_{h}}{L\left(L-1\right)} \end{array}$$

(11) $$\begin{array}{l} F_{E}=\frac{\sum_{i=1}^{{Count}_{e}}{Posi}_{e}}{L\left(L-1\right)} \end{array}$$

(12) $$\begin{array}{l} F_{C}=\frac{\sum_{i=1}^{{Count}_{c}}{Posi}_{c}}{L\left(L-1\right)} \end{array}$$

(13) $$\begin{array}{l} F_{Max\text{\_}E}=\frac{Max\text{\_}{Length}_{e}}{L} \end{array}$$

(14) $$\begin{array}{l} F_{Max\text{\_}H}=\frac{Max\text{\_}{Length}_{h}}{L} \end{array}$$

Where *Count*_*h*_, *Count*_*e*_, and *Count*_*c*_ are the total number of the *H*, *E*, and *C* in *S*_*structure*_; *Posi*_*h*_, *Posi*_*e*_, and *Posi*_*c*_ represent the position index of *H*, *E*, and *C* respectively; *Max*_*Length*_*e*_ and *Max*_*Length*_*h*_ are the largest numbers of continuous *E* and *H*. Then, transfer *S*_*structure*_ to the segment sequence *S*_*segment*_ by deleting coil states and continuous *H* and *E* are treated as segment *H* and segment *E*, and expressed in terms of α and β, respectively (Zhang et al., 2011). For instance, structure sequence EECCCHHHEEECHHHEECCEE can be transfer to segment sequence βαβαββ. Then, frequency of segment βαβ in *S*_*segment*_ is defined as a feature and formulated as (Wei et al., 2015)

(15) $$\begin{array}{l} F_{frequency\text{\_}\beta \alpha \beta }=\frac{{Count}_{\beta \alpha \beta }}{L-2} \end{array}$$

where *Count*_βαβ_ is the total number of segment βαβ.

(4) Extract 3 global and 24 local structural features from the structure probability matrix. Structure probability matrix *M*_*probability*_ also is the profile of PSI-PRED on sequence, which can be represented by (Wei et al., 2015)

(16) $$M_{probability}={\left|\begin{array}{ccc} pro_{1,1} & pro_{1,2} & pro_{1,3}\\ pro_{2,1} & pro_{2,2} & pro_{2,3}\\ \vdots & \vdots & \vdots \\ pro_{i,1} & pro_{i,2} & pro_{i,3}\\ \vdots & \vdots & \vdots \\ pro_{L,1} & pro_{L,2} & pro_{L,3} \end{array}\right|}_{L\times 3}s.t.1\leq i\leq L$$

where *pro*_*i*,1_, *pro*_*i*,2_, and *pro*_*i*,3_ are the probability values of amino acid residue in sequence to predict as secondary structure states of “C,” “H,” and “E,” respectively. Thus, this matrix has *L* rows. Three global structural features are calculated by averaging each column value as follows (Wei et al., 2015)

(17) $$F_{pro\_global}=\left\{\frac{\sum_{i=1}^{L}Pro_{i,1}}{L},\frac{\sum_{i=1}^{L}Pro_{i,2}}{L},\frac{\sum_{i=1}^{L}Pro_{i,3}}{L}\right\}$$

Then *M*_*probability*_ is divided into λ sub-matrices and three global structural features as Exp. (17) are calculated separately. Finally, obtain λ × 3 features. We chose the λ = 8 in this study, which are represented as (Wei et al., 2015)

(18) $$\begin{array}{l} F_{pro\_local}=\{f_{pro_{local_{1}}},f_{pro_{local_{2}}},\ldots ,f_{pro\_local\_i},\ldots ,f_{pro\_local\_8}\}\\ s.t.1\leq i\leq 8 \end{array}$$

where *f*_*pro*_*local*_*i*_ express three values consisting of the average of each column value in the submatrix. Therefore, there are 8 × 3 elements in vector *F*_*pro*_*local*_.

Finally, the above features are combined in the following order to form the 473D feature, which is represented as (Wei et al., 2015):

(19) $$\begin{array}{c} \{F^{\prime }{\;}_{1-gram},F^{\prime }{\;}_{2-gram},F_{pssm},F_{H},F_{C},F_{E},F_{Max\_H},F_{Max\_E},\\ F_{frequency\_\beta \alpha \beta },F_{pro\_local}\;,\;F_{pro\_global}\} \end{array}$$

### Feature Selection

Feature selection aims to select a subset of features to improve the generalization capacity of the learning models. The Max-Relevance-Max-Distance algorithms (MRMD) (Zou et al., 2016b) was utilized for feature selection. It has two steps—ranking features and selecting optimal feature sets.

First, calculate the MRMD score of each feature vector. The MRMD score of a feature vector consists of a relevant value and a distance value. The former indicates the relevant value of a feature and target class vector, and it equals the Pearson correlation coefficient (Xu and Deng, 2018) between the feature and target class vector, which is calculated using the following formula (Zou et al., 2016b):

(20) $$RV_{i}=PCC(f_{i},c)=\frac{\sum_{k=1}^{N}\left(f_{i}\left(k\right)-\bar{f_{i}}\right)(c\left(k\right)-\bar{c})}{\sqrt{{\sum_{k=1}^{N}\left(f_{i}\left(k\right)-{\bar{f}}_{i}\right)}^{2}}\sqrt{{\sum_{k=1}^{N}\left(c\left(k\right)-\bar{c}\right)}^{2}}}$$

where ${\bar{f}}_{i}=1/N(\sum_{k=1}^{N}f_{i}\left(k\right)$ and similarly $\bar{c}=1/N(\sum_{k=1}^{N}c\left(k\right)$. *f*_*i*_ is the *i*th feature vector and *c* is the target class vector, which consists of 0 and 1 in this study. *RV*_*i*_ is relevant value of *i*th feature vector and equals to Pearson correlation coefficient between *f*_*i*_ and *c*. *N* is the number of elements in a feature vector, and equals the total number of samples in the dataset. *f*_*i*_(*k*) denotes the *k*th element of feature *f*_*i*_.

The distance value is a measurement of feature redundancy and is calculated by the Euclidean distance function as follows (Zou et al., 2016b; Dong et al., 2019):

(21) $$DV_{i}=\frac{1}{N}\sum_{j=1}^{N}ED(f_{i},f_{j})$$

where *DV*_*i*_ is the distance value of the *i*th feature vector. *ED*(*f*_*i*_, *f*_*j*_) denotes the Euclidean distance of *i*th and *j*th feature vector and is formulated by (Zou et al., 2016b):

(22) $$ED(f_{i},f_{j})=\sqrt{{\sum_{k=1}^{N}\left(f_{i}\left(k\right)-f_{j}(k)\right)}^{2}}$$

Based on Equations (20) and (21), the MRMD score of feature *f*_*i*_ is defined as (Zou et al., 2016b)

(23) $$MRMD\_\;score_{i}=\;RV_{i}+\;DV_{i}$$

Inverse sorting of feature set (19) using MRMD score to obtain new feature set *F*′, which is represented as

(24) $$F^{\prime }=\;[f_{1}\prime ,f_{2}\prime ,\cdot \cdot \cdot ,f_{n-1}\prime ,f_{n}\prime ]$$

Candidate subsets were constructed by adding from features in *F*′ one-by-one each time in ranking order, and can be expressed as: $\left[f_{1}\prime \right],\;\left[f_{1}\prime ,f_{2}\prime \right],\;\left[f_{1}\prime ,f_{2}\prime ,f_{3}\prime \right]\ldots f_{1}\prime ,f_{2}\prime \ldots f_{n-1}\prime ,f_{n}\prime$. Then, the above subsets were fed into random forest and construct models separately. Among them, a subset of the best performance is selected as the optimal feature set.

### Support Vector Machine

Support Vector Machine (SVM) has been widely used in the bioinformatics fields and has performed excellently (Cao et al., 2014; Stephenson et al., 2019). SVM is a method based on the theory of Vapnik–Chervonenkis Dimension (Vapnik et al., 1994) (VC Dimension) and structural risk minimization. SVM maps low-dimensional data to high-dimensional space and uses hyperplane to segment different labeled data. In this study, we chose the toolbox LIBSVM 3.21 (Chang and Lin, 2011) to execute the SVM. It can be downloaded from https://www.csie.ntu.edu.tw/~cjlin/libsvm/. The default kernel function—the radial basis function (RBF) was adopted, and python program grid.py in the toolbox LIBSVM 3.21 was used to search the optimized value of the penalty constant *C* and the kernel width parameter γ. To correctively evaluate a model with an unbalanced data set, the official website provides a tool that enables LIBSVM to conduct cross-validation with respect to other criteria, including F-score, AUC (Area Under Curve), precision, recall, and more (this tool is available at https://www.csie.ntu.edu.tw/~cjlin/libsvmtools/eval/index.html).

### Proposed Classifier Flowchart

**Figure 1:**
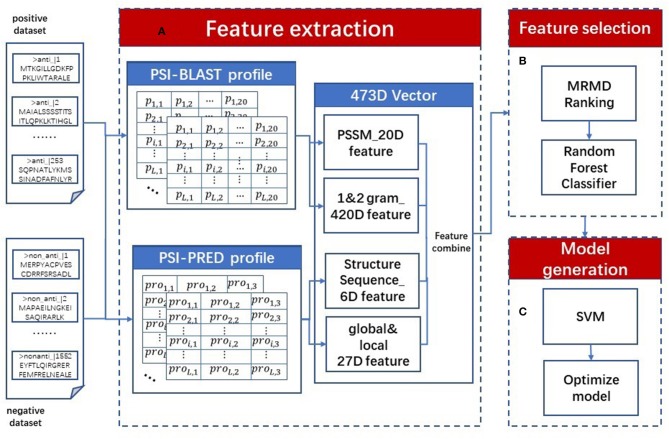
AOPs-SVM flowchart. The original dataset (positive and negative dataset) is processed in three phases. (A) In the feature extraction phase, two types of profiles are constructed using the PSI-BLAST and PSI-PRED programs. Then, 473D discrete vectors are generated by combining evolutionary information and secondary processing feature information, including 20D PSSM features, 20D 1-g, 400D 2-g features, 6D secondary structure sequence features and 27D global and local structural features. (B) In feature selection phase, ranking the 473D features by MRMD score and selecting optimal feature set by Random Forest. (C) At last, in model generation phase the optimal feature set is fed into SVM to generate the AOPs-SVM model and optimize it via a grid search.

We proposed a sequence-based classifier using a support vector machine named AOPs-SVM; a flowchart is presented in Figure 1. The AOPs-SVM procedure consists of three phases: (1) feature extraction, (2) feature selection, and (3) model generation. In phase (1), the input protein sequences are processed by the PSI-BLAST and the PSI-PRED programs. The resulting profiles generate 473-dimension (473D) discrete vectors, including evolutionary information and secondary structure information. Then, in phase (2), these 473D vectors were fed into the MRMD method to rank and select the optimal feature set by random forest. In the model generation phase, the SVM was applied to generate a model on the optimal feature set. Lastly, this model was optimized by selecting the optimal value for the penalty constant *C* and the kernel width parameter γ by grid search in terms of F1 score.

### Measurement

There are three kinds of evaluation methods commonly used in bioinformatics fields: an independent test, a k-fold cross validation and a jackknife test (Wei et al., 2017a,b, 2018; Chen et al., 2018; Liu et al., 2018a,b; Ding et al., 2019; Lv et al., 2019; Yang et al., 2019b). In a jackknife test, each sample is tested by the model, which is trained by all other samples. In this study, we applied the jackknife test, as it is the most rigorous and least arbitrary method. Considering the unbalanced dataset used, sensitivity (Sn), specificity (Sp), accuracy (Acc), and Mathew's correlation coefficient (MCC) were employed as the evaluation metrics. The F1 score was used as the criterion for optimizing the model.

(25) $$Sn=\frac{TP}{TP+FN}$$

(26) $$Sp=\frac{TN}{TN+FP}$$

(27) $$Acc=\frac{TN+TP}{TN+FP+FN+TP}$$

(28) $$F1=2\times \frac{\frac{TP}{TP+FP}\times \frac{TP}{TP+FN}}{\frac{TP}{TP+FP}+\frac{TP}{TP+FN}}\;$$

(29) $$MCC=\frac{TN\times TP-FP\times FN}{\sqrt{\left(TP+FP)\times (FN+TN)\times (TP+FN)\times (TN+FP\right)}}$$

where TP, FP, FN, and TN indicate true positive, false positive, false negative, and true negative, respectively. In addition, Area Under Curve (AUC) is an important metric and accurately measures the overall performance of the model. It is the value of the area enclosed by the receiver operating characteristic curve (ROC curve) and the two coordinate axes. The ROC curve is a continuous line plotted by (1 − *Sp*) as X-coordinate and *Sn* as the Y-coordinate. The larger the AUC value, the better the performance of the model.

## Results and Discussion

### Determination of Parameters

There are two groups of parameters that have to be determined in the proposed classifier: the parameters associated with the random forest in the feature selection phase, and the parameters associated with the optimizing SVM in the model generation phase. The random forest parameters were initialized as follows: the number of trees was set to 100; the number of features to use in random selection was set to 0; the seed for the random number generator was set to 1; and the maximum depth of the tree was 0 for unlimited. The grid.py parameter selection tool was applied to evaluate the SVM in the model generation phase with *F*1 criteria under jackknife test. It involved searching the optimized value to penalty constant parameter *C* and the kernel width parameter γ. logarithmic function was adopted and set the searching range of $\log_{2}^{c}$ as {−5,15} with step of 0.5, similarly, searching range of $\log_{2}^{\gamma }$ is {3,−15} with a step of −0.5.

### Performance of the Proposed Classifier

**Figure 2:**
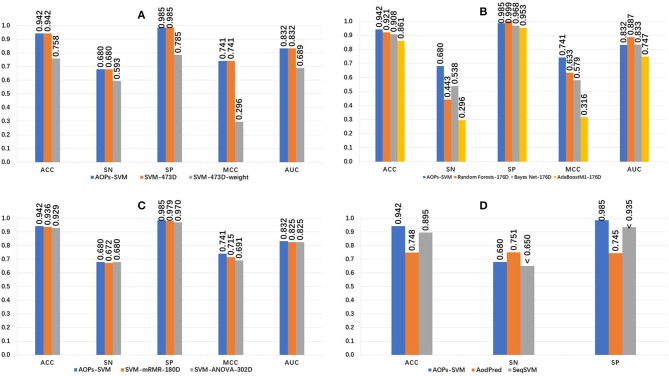
Performance comparison of the AOPs-SVM and other classifiers. **(A)** Compares other SVM models generated on the original feature set (473D). SVM-473D and SVM-473D-weight are the classifiers that the SVM trained on the original feature set in straight and weighted manner (negative: positive = 1: 6). **(B)** Comparing with three other traditional classifiers on optimal feature set (176D). RandomForest-176D, BayesNet-176D, and AdaBoostM1-176D are RandomForest, BayesNet and AdoBoostM1 on optimal feature set, respectively. **(C)** Comparing with other SVM models based on optimal feature set generated by ANOVA and mRMR respectively. ANOVA, mRMR generated 302D and 180D optimal feature set, respectively. **(D)** Comparing with state-of-the art methods. “ < ” denotes that Sn and SP of SeqSVM are <0.65 and 0.935, respectively.

The 473D features were extracted in the feature extraction phase and ranked by MRMD score. The random forest method was applied and a 176D optimal feature set was selected. Then, this optimal feature set was fed into the SVM model and optimized to generate the proposed AOPs-SVM classifier. To evaluate the performance of the proposed classifier, we conducted a series of comparisons, the results of which are presented in Figure 2.

The proposed AOPs-SVM classifier achieved 94.2% in ACC, 0.68 in sensitivity, 0.985 in specificity, 0.741 in MCC, and 0.832 in AUC. As seen in Figure 2A, the AOPs-SVM achieves the same performance with the SVM-473D, which is much better than the SVM-473D-weight. This demonstrates that the feature selection phase effectively solves for data redundancy when the feature set shrinks from 473D to 176D. In Figure 2B, although the random forest, Bayes Net, and AdoBoostM1 all achieve high specificity scores, they are inefficient in sensitivity, while two are even lower than random classification. This shows that the SVM produces a more balanced result on an optimal feature set compared to three other candidate classifiers. Figure 2C shows that AOPs-SVM is superior to SVM-mRMR-180D and SVM-ANOVA-302D. This result demonstrates that the MRMD algorithm not only results in a lower dimension (176D), but also retains the important features in the optimal feature set. In Figure 2D, the performance of the proposed classifier is compared to the AodPred (Feng et al., 2016) and SeqSVM (Xu et al., 2018) in term of sensitivity, specificity, and accuracy. The AOPs-SVM is slightly lower than AodPred for sensitivity. However, it outperforms the other two classifiers in specificity and accuracy.

### Feature Contribution and Importance Analysis

**Figure 3:**
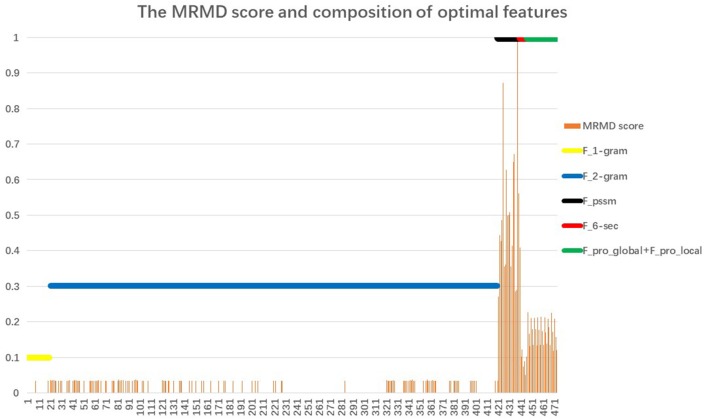
The MRMD score and composition of the optimal feature set. The X-coordinate corresponds to 473 features; the Y-coordinate is the value of the MRMD score and participation rate. The orange vertical line represents the MRMD score of the 176 optimal feature set. Just as with the original feature set, the optimal feature set also consists of 6 feature types. The six horizontal lines represent the participation rates of each feature: 20D 1-gram feature (yellow); 400D 2-g feature (blue); 20D feature from PSSM (black); 6D secondary structure feature (red); and 27D global and local feature (green). We defined the participation rate as equal to the number of each feature type in the 176 feature set divided by the total number of corresponding features. For example, the 6D secondary structure feature (red) are all selected for inclusion in the optimal feature set, so the participation rate is 1.

Section Performance of the Proposed Classifier noted that the proposed AOPs-SVM classifier was trained on the optimal feature set (176D), and achieved the same performance as the SVM trained on the original feature set (473D). This demonstrates that the optimal feature set retained the important features. The MRMD score and feature composition of the optimal feature set (176D) are shown in Figure 3.

When comparing the six horizontal lines, the 20D feature from the PSSM, the 6D secondary structure features, and the 27D global and local features corresponding to *F*_*pssm*_, {*F*_*H*_, *F*_*C*_, *F*_*E*_, *F*_*Max*_*H*__, *F*_*Max*_*E*__, *F*_*frequency*_βαβ__} and {*F*_*pro*_*local*__, *F*_*pro*_*global*__} of Equation (19), respectively, achieve the highest participation rate with reaching 100%. The latter two features come from PSI-PRED profile. It indicates that secondary structure information extracted from PSI-PRED profiles highly contributes to the antioxidant protein identification task. Analysis from the view of combining MRMD score and participation rate, the 20D feature from matrix PSSM, that is *F*_*pssm*_ in Equation (4), obtains the highest 20 MRMD scores and all of them appear in the 176D optimal feature set. It indicates that 20 evolutionary features in *F*_*pssm*_ have the most relevance to the target classification, but have the least redundant information. Therefore, we can conclude from a bioinformatics perspective that *F*_*pssm*_ can be selected as an important marker for identifying antioxidant proteins. These 20 *F*_*pssm*_ features' MRMD scores are shown in Table 1, where the odd-numbered rows are the order number of features slashed by the corresponding mutating residue. The even-numbered rows are the MRMD scores.

**Table 1:** MRMD score of *F*_*pssm*_.

**Order**	**F421/A**	**F422/R**	**F423/N**	**F424/D**	**F425/C**	**F426/Q**	**F427/E**	**F428/G**	**F429/H**	**F430/i**
MRMD score	0.271886	0.443691	0.427483	0.486034	0.870973	0.355192	0.360546	0.628175	0.499345	0.500683
**Order**	**F431/L**	**F432/K**	**F433/M**	**F434/F**	**F435/P**	**F436/S**	**F437/T**	**F438/W**	**F439/Y**	**F440/V**
MRMD score	0.508348	0.355434	0.414661	0.649391	0.672217	0.286041	0.289247	1	0.560823	0.408259

## Conclusions

In this paper, we proposed a novel approach for identifying antioxidant proteins, and constructed a classifier called AOPs-SVM. The 473D discrete features, including evolutionary information and secondary structure information, were extracted from the training set. To eliminate redundant data, the MRMD algorithm was applied and the 176D optimal feature set was obtained. Then, the AOPs-SVM was generated by an SVM model based on the optimal feature set. Experimental results show that the proposed classifier is superior to other classifiers, including state-of-the art methods. In addition, we analyzed the contribution and composition of the optimal feature set using bioinformatics techniques. In the future, we will attempt to improve the performance achieved in this study by (1) searching and combining potential and significant features, as well as by using a more effective feature selection approach (Yu et al., 2018); and (2) adopting other classifying algorithms, such as extreme learning (Li et al., 2019a) and deep learning (Cao et al., 2017; Long et al., 2017; Conover et al., 2019; Hou et al., 2019; Zhang et al., 2019; Zou et al., 2019), etc.

## Data Availability

Publicly available datasets were analyzed in this study. This data can be found here: http://server.malab.cn/AOPs-SVM/data.jsp.

## Author Contributions

CM, QZ, and SJ wrote the paper, participated in the research design, and developed the web server. LW and FG participated in preparation of the manuscript. CM, SJ, LW, FG, and QZ read and approved the final manuscript.

### Conflict of Interest Statement

The authors declare that the research was conducted in the absence of any commercial or financial relationships that could be construed as a potential conflict of interest.
